# A novel prolixicin identified in common bed bugs with activity against both bacteria and parasites

**DOI:** 10.1038/s41598-024-64691-4

**Published:** 2024-06-15

**Authors:** Sanam Meraj, Arshvir Singh Dhari, Emerson Mohr, Carl Lowenberger, Gerhard Gries

**Affiliations:** Department of Biological Sciences, Simon Fraser University, Burnaby, BC V5A1S6 Canada

**Keywords:** Bed bugs, Prolixicin, Glycine-rich antimicrobial peptides, Toll and IMD humoral innate immunity, *Trypanosoma cruzi*, Chagas disease, Biochemistry, Computational biology and bioinformatics, Drug discovery, Evolution, Genetics, Immunology, Microbiology, Molecular biology, Diseases, Medical research

## Abstract

The hematophagous common bed bug, *Cimex lectularius*, is not known to transmit human pathogens outside laboratory settings, having evolved various immune defense mechanisms including the expression of antimicrobial peptides (AMPs). We unveil three novel prolixicin AMPs in bed bugs, exhibiting strong homology to the prolixicin of kissing bugs, *Rhodnius prolixus,* and to diptericin/attacin AMPs. We demonstrate for the first time sex-specific and immune mode-specific upregulation of these prolixicins in immune organs, the midgut and rest of body, following injection and ingestion of Gr+ (*Bacillus subtilis*) and Gr– (*Escherichia coli*) bacteria. Synthetic CL-prolixicin2 significantly inhibited growth of *E. coli* strains and killed or impeded *Trypanosoma cruzi*, the Chagas disease agent. Our findings suggest that prolixicins are regulated by both IMD and Toll immune pathways, supporting cross-talk and blurred functional differentiation between major immune pathways. The efficacy of CL-prolixicin2 against *T. cruzi* underscores the potential of AMPs in Chagas disease management.

## Introduction

Antimicrobial peptides (AMPs) are essential components of immune defense mechanisms in insects, serving as important contributors to the coevolutionary race between insect hosts and pathogens^1–6^. AMPs contribute to the elimination of microbial pathogens and, together with physical and cellular immune responses, are vital components of the multi-faceted innate immune responses of insects^1–6^. Immune responses have three sequential stages: (1) recognition of non-self entities, (2) activation of signal transduction pathways, and (3) production of effector molecules that eliminate infectious agents. Activation of the immune system is dependent upon the fundamental ability to recognize non-self entities which, in turn, activates cellular and humoral responses in parallel^4,7–9^.

Recognition of non-self entities such as microbes entails interactions of peptidoglycan recognition proteins (PGRPs) and Gram-negative (Gr–) binding proteins (GNBPs) with microbial surface molecules^9–12^. Toll and IMD are two principle immune pathways that are triggered in response to bacterial infections, ultimately resulting in the expression of AMPs in multiple tissues. Initial studies in the vinegar fly *Drosophila melanogaster* suggested that the Toll pathway is activated by Gr+ bacteria and fungi, whereas the IMD pathway is activated by Gr– bacteria. We now know that there is significant plasticity and cross talk among multiple immune pathways, breaking the initial paradigm^13^. Ultimately, the activation of immune-related pathways induces the expression of various effector molecules including AMPs^14–17^. AMPs are synthesized mainly in the fat body and released into the hemolymph or gut lumen by gastrointestinal epithelial cells, but are also produced in the midgut, hemocytes, and other insect tissues and organs^16–19^.

The common bed bug, *Cimex lectularius* (CL), is a global blood-feeding pest that has plagued humanity for centuries^20–22^. The question whether bed bugs can transmit agents of human infectious diseases remains of great interest^22–25^. Many pathogens have been isolated from bed bugs^22,23, 25, 26^, and various parasites have been transmitted by bed bugs under laboratory conditions^23,27–29^. One such parasite is *Trypanosoma cruzi*, the etiological agent of Chagas disease, which causes premature heart failure of afflicted humans in the Americas. In field settings, however, bed bugs are not known to transmit *T. cruzi* or other pathogens. This is in contrast to many other hematophagous arthropods that serve as biological vectors for human and animal pathogens^22,23, 25^. It has been hypothesized that some components of the bed bugs’ innate immune responses, including AMPs, prevent the establishment and replication of pathogens and their transmission to new hosts. Moreover, since 2020, the World Health Organization has assigned key roles to AMPs in the fight against neglected tropical diseases, including Chagas disease^30,31^.

The bed bug genome reveals strong candidates for key components of the Toll, IMD, Jak/STAT, apoptosis, autophagy, and RNAi related immune pathways^20^. Interestingly, bed bugs, and many other hemimetabolous insects, have fewer components of the IMD pathway than holometabolous insects^20^. Similarly, only defensins—attacin/diptericin-like AMPs (containing a glycine-rich domain)—have been identified in the bed bug genome^20^. The bed bug's immune system is considered unique due to adaptations prompted by their distinct reproductive strategy, traumatic insemination, wherein males inject sperm directly into the female's body cavity, increasing infection risks^20,32–36^.

Prolixicin is a glycine-containing AMP that was identified first in the kissing bug *Rhodnius prolixus,* and has strong homology with members of the attacin/diptericin AMP superfamilies^16,37^. In contrast to other glycine-rich AMPs, prolixicin has a shorter and simpler amino acid structure, lacking the proline-rich sequence (P-domain) and containing only a signal and a glycine-rich moiety (G-domain) in its C-terminus^16,37^. Diptericins are glycine-rich peptides. It is hypothesized that a duplication event added a second glycine-rich domain to diptericins, resulting in the formation of attacins^38^. Attacins contain a signal peptide, a P-domain ending with a conserved R-X-(R/K)-R, an N-domain, and a C-domain which is also referred to as G1- or G2-domain^38^. In contrast to diptericins and attacins, prolixicins lack the conserved furin cleavage site in the canonical sequence R-X-(R/K)-R which causes the separation of the signal- and pro-peptide region, and the cleavage to release the mature/active peptide^16^.

AMPs have been described primarily with respect to their physical structure and the types of organisms they kill (bacteria, fungi, parasites)^1,18^. Mature proline- and glycine-rich AMPs, such as diptericins, are active predominantly against Gr– bacteria^17^, whereas prolixicins—like cecropins—are active against both Gr– and Gr+ bacteria^16^. The activity of attacins/diptericins is thought to be linked to specific chemo-physical properties (e.g., positive charge, abundance of hydrophobic residues) of certain sequence sections that may interact with the bacterial membrane and express antimicrobial activity^39^. Attacins have been shown to increase the permeability of the outer bacterial membrane of *Escherichia coli,* and to inhibit the synthesis of outer-membrane proteins^40^. The structural and chemical properties that contribute to AMP activity of glycine-rich peptides need to be investigated.

Here, we characterized two novel prolixicins in bed bugs (identified in a transcriptome of immunized bed bugs; SM unpubl.), and compared their molecular features, structural properties, and phylogenetic relatedness with those of other glycine-rich insect AMPs (e.g., diptericins, attacins). We studied the expression of prolixicins in the midgut and rest of body (RoB) in bed bugs after ingestion of bacteria-laced blood or injection of bacteria into the bed bugs’ hemocoel. Using comparative transcriptomics and quantitative real-time PCR (qPCR), we investigated the effects of bacterial type and mode of entry (ingestion *vs*. injection), as well as bed bug sex and tissue, on the up-regulation of these prolixicins in bed bugs. Finally, we tested synthetic CL-prolixicin2 for its antimicrobial and antiparasitic properties against a range of microbes and the Y strain of *T. cruzi* which had been isolated from patients with Chagas disease. Our study adds to current knowledge about the diversity of AMPs expressed in bed bugs, and it provides a foundation for further research on the humoral immunity of bed bugs in response to pathogenic and parasitic infections. Altogether, our data help explain why bed bugs do not transmit parasites and pathogens to their vertebrate hosts.

## Results

### Structural, functional and phylogenetic analyses of CL-prolixicins (Figs. 1, 2, 3)

**Figure 1:**
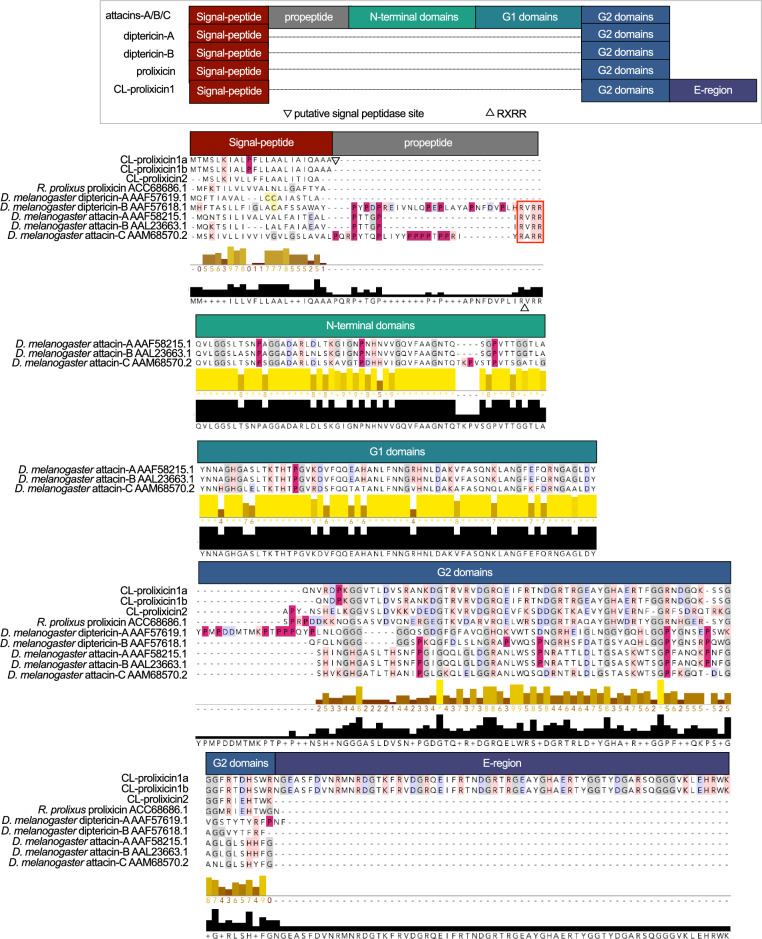
Comparison of structural features of glycine-rich peptides in bed bugs and other select insects. Sequences were aligned using both MUSCLE (multiple sequence comparison by log expectation; https://www.ebi.ac.uk/Tools/msa/muscle/) and multiple sequence alignment (MSA). Conserved prolines are crimson-highlighted, positively charged side groups (basic residues; lysine (K), arginine (R), histidine (H)) are red-highlighted, negatively charged side groups (acidic residues; glutamate (E), aspartate (D)) are blue-highlighted, and cysteines are yellow-highlighted. The predicted signal-peptide, pro-peptide, and mature-peptide regions are indicated in boxes above MSA, and the cleavage sites between these regions are indicated by upwards and downwards arrows, as indicated in the figure legend. Conserved glycines are grey-highlighted.

**Figure 2:**
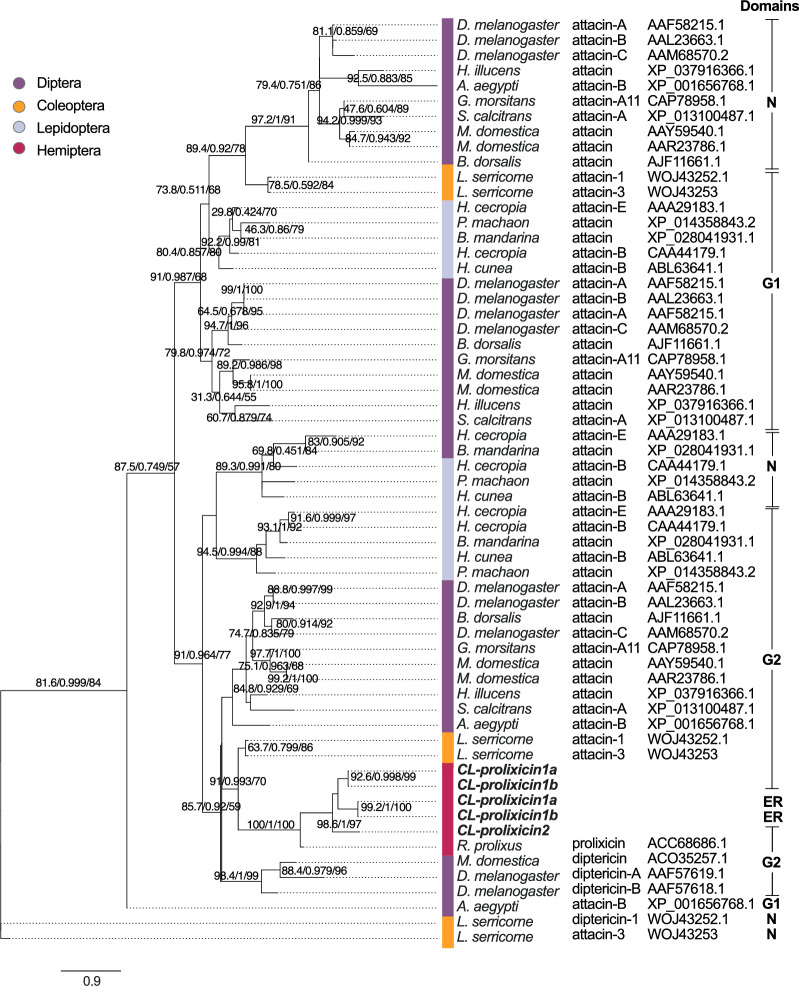
Phylogeny of domains of CL-prolixicins identified in bed bugs in comparison with glycine-rich antimicrobial peptides from other insect orders. The bed bug prolixicins are most closely aligned with the prolixicin of the kissing bug *Rhodnius prolixus*. Glycine-rich sequences were aligned with MUSCLE (https://www.ebi.ac.uk/Tools/msa/muscle/), and alignments were used to build phylogenetic trees using iqtree-2.0-rc2 with substitution models PMB + F + G4. The tree was finalized using FigTree software v. 1.4.4 (http://tree.bio.ed.ac.uk/software/figtree/). Phylogenetic testing included 10,000 replicates of Ultrafast bootstrap (UFBoot), Bayesian inference, and maximum likelihood analyses represented on each branch to provide support for tree branches. The tree scale indicates 0.9 substitutions per site, providing a measure of evolutionary distance.

**Figure 3:**
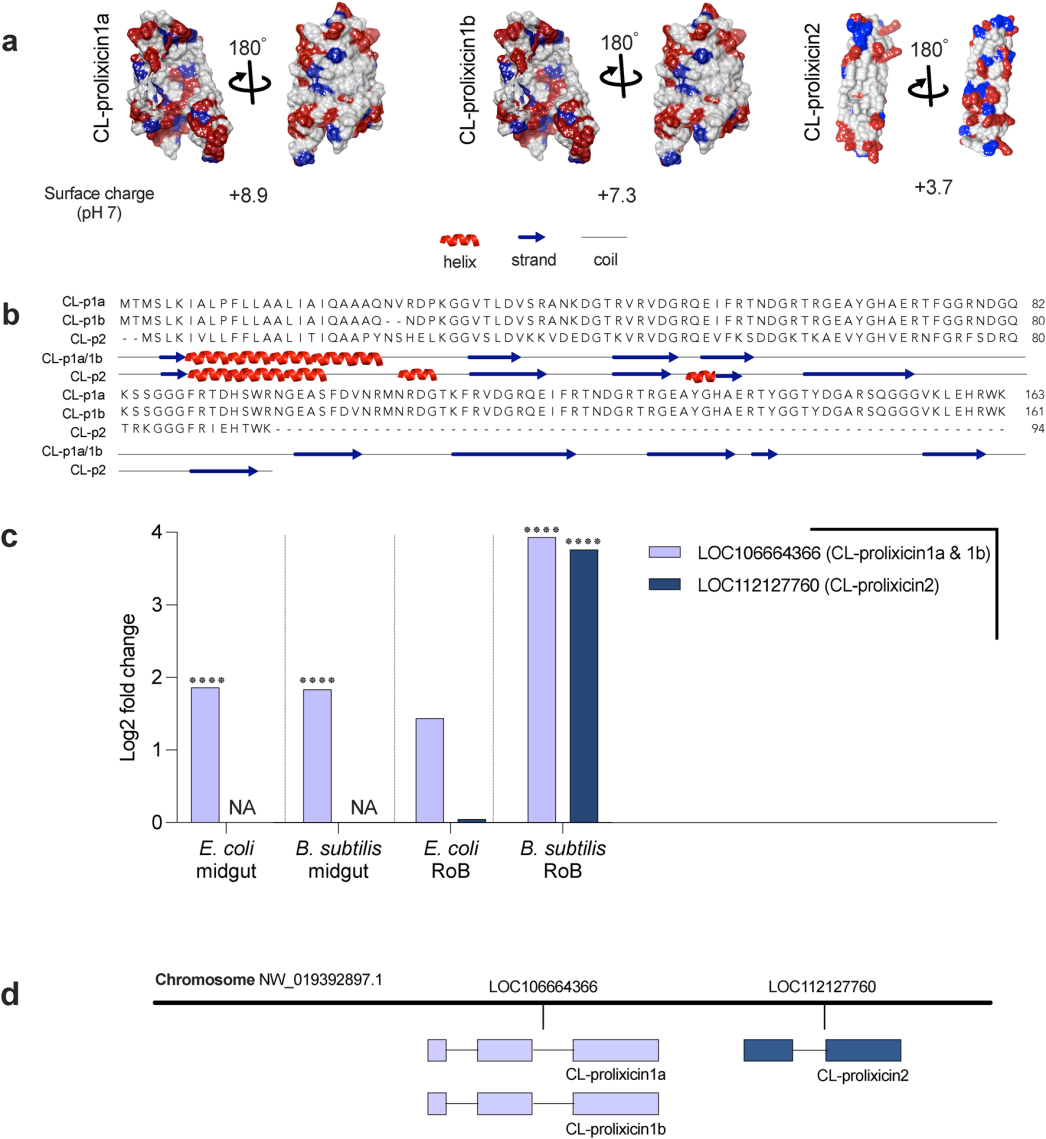
Comparative transcriptomics and structural analysis of the antimicrobial peptides CL-prolixicin1a & 1b and CL-prolixicin2 in bed bugs. (a) Predicted structures, structural surface characteristics, and pH of CL-prolixicin1a & 1b, and of CL-prolixicin2, using I-TASSER (Iterative Threading ASSEmbly Refinement), visualized using UCSF Chimera. Positively and negatively charged residues are indicated in blue and red, respectively. (b) Secondary structure prediction of the sequences of CL-prolixicin1a (CL-p1a) & 1b (CL-p1b), and of CL-prolixicin2 (CL-p2) predicted by I-TASSER. (c) Changes in gene expression in bed bug midgut and RoB tissues (rest of body containing bodies minus heads and midgut tissues) were quantified after bed bugs ingested sterile blood or blood infected with the Gram-positive bacterium *Bacillus subtilis* or the Gram-negative bacterium *Escherichia coli*. The Wald test was used to generate p-values and Log2 fold changes. An asterisk indicates statistically significant changes in gene expression levels (adjusted p-values < 0.05). (d) Map of genes expressing prolixicins in bed bugs. Genes are located on the Chromosome NW_019392897.1. CL-prolixicin1a & 1b (expressed from LOC106664366) and CL-prolixicin2 (expressed from LOC122127760) are in close proximity. Predicted exons are shown as boxes, and introns are illustrated by black lines. The direction of transcription is from left to right.

Three unknown attacin/diptericin-like bed bug peptides containing a predicted attacin-C domain (cl04253) were characterized. Similar to the prolixicin of *R. prolixus,* CL-prolixicin1a (XP_014245530.1), CL-prolixicin1b (XP_014245531.1), and CL-prolixicin2 (XP_024084959.1) all lack the P-domain. The mature CL-prolixicin2 has a molecular weight of ~ 9 kDa similar to that of insect diptericins. However, the mature CL-prolixicin1a & 1b have a molecular weight of approximately 15 kDa due to an additional glycine-rich stretch of sequences (E-region) at their terminal region, which is not conserved in attacins and diptericins (Fig. 1, E-region). This sequence stretch had no predicted antimicrobial, antiviral, or antifungal activity despite its ~ 60% homology with the prolixicin of *R. prolixus.*

Using multiple sequence alignment, we compared the conserved domains of glycine-rich peptides, including the signal-peptide, pro-peptide, N-terminus, G1- and G2-domains, in bed bug CL-prolixicins with those of diptericins, prolixicin and attacins previously identified in *R. prolixus* and *D. melanogaster* (Fig. 1). The signal-peptide of CL-prolixicin1a & 1b is located between position 23–24 (AA-Q), and between position 19–20 (A-A) in CL-prolixicin2. No arginine/lysin pro-peptide cleavage site was detected in any CL-prolixicin. Multiple sequence alignment of bed bug CL-prolixicins, the domains of glycine-rich AMPs in *R. prolixus,* and the attacin domains of *D. melanogaster* attacins and diptericin further revealed close phylogenetic relationships between the bed bug CL-prolixicins, and the *R. prolixus* prolixicin (Fig. 2). The surface charge of CL-prolixicin1a (8.9), CL-prolixicin1b (7.3), and CL-prolixicin2 (3.7) is comparable to the surface charge of other glycine-rich peptides^39^ (Fig. 3a,b). We have also predicted AMP activity of these prolixicins (Supplemental Table S2).

### Comparative transcriptomics of CL-prolixicins after ingestion of bacteria

Comparative transcriptomics of male bed bugs revealed a significant upregulation of CL-prolixicins in midgut and RoB samples (Fig. 3c). CL-prolixicin1a & 1b mRNA (LOC106664366) was significantly upregulated in (*i*) the midgut following ingestion of either *E. coli* or *B. subtilis*, and (*ii*) the RoB after ingestion of blood infected with *B. subtilis*. CL-prolixicin2 mRNA (LOC122127760) was expressed only in the RoB after ingestion of *B. subtilis* infected blood. CL-prolixicin1a and CL-prolixicin1b (expressed from LOC106664366) are situated in close proximity to CL-prolixicin2 (expressed from LOC122127760) on the same chromosome (NW_019392897.1) (Fig. 3d).

### Distinct gene expression of CL-prolixicin1 in midgut and RoB of male and female bed bugs after bacterial ingestion or injection and evaluation of effects of bed bug sex, and mode of immune challenge (Fig. 4)

**Figure 4:**
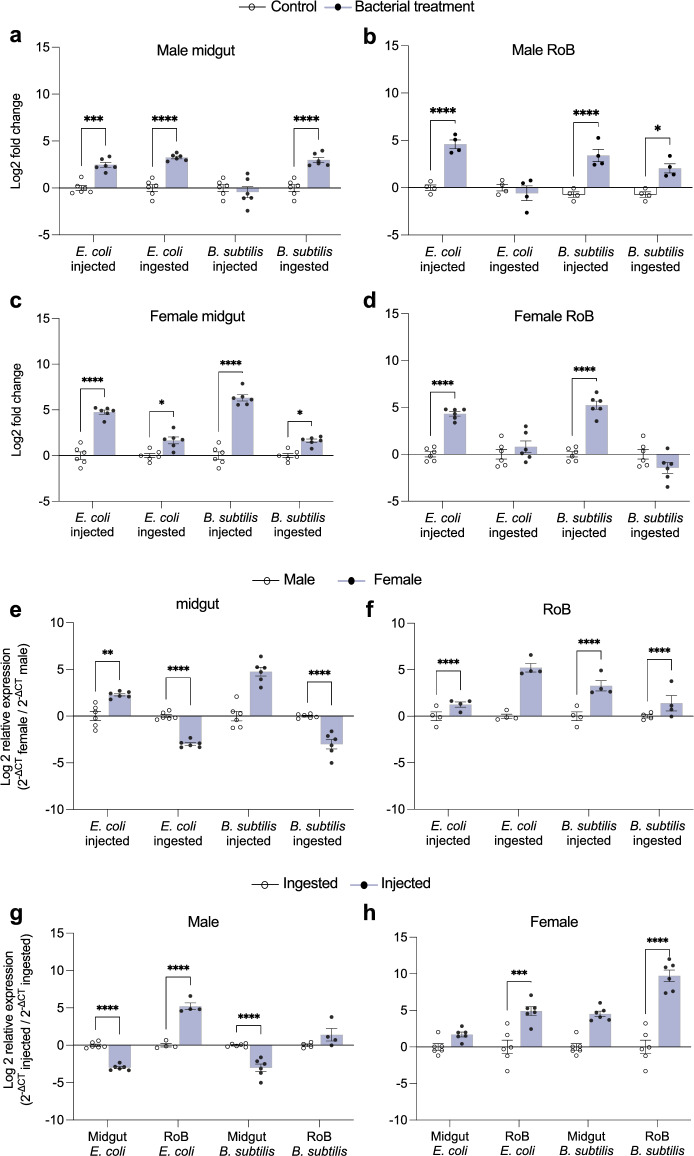
Tissue- and sex-dependent expression of CL-prolixicin antimicrobial peptides by bed bugs following bacterial injection & ingestion. (a–d) Changes in expression levels of CL-prolixicin1a & 1b (LOC106664366) in the midgut and RoB (Rest of Body: bodies minus heads and midgut tissues) of male and female bed bugs 12 h after intrathoracic injection or ingestion of Gram-negative bacteria (*Escherichia coli* K12/D31) or Gram-positive bacteria (*Bacillus subtilis* ATCC 6633). White bars represent data obtained from control bugs that were injected with phosphate buffer saline (PBS) or that ingested sterile blood, evaluated using the ΔΔCT method^64,65^. (e,f) Effect of bed bug sex on changes in expression levels of CL-prolixicin1a & 1b (LOC106664366) in the midgut and RoB. (g,h) Comparison of the mode of infection—bacterial ingestion or injection—on changes in expression levels of CL-prolixicin1a & 1b (LOC106664366) in male and female bed bugs. The relative expression of CL-prolixicin1a & 1b was evaluated using the ΔΔCT method^64,65^. Data from males were used as the second calibrator and were arbitrarily set to 1 (subpanels e,f); the fold-changes of expression in females are shown as purple bars. The effect of bacterial ingestion *versus* bacterial injection was compared using the formula 2^−ΔCt^ injected/2^−ΔCt^ ingested (subpanels g,h). The ingestion-sample data (white bars) representing the calibrator were arbitrarily set to 1, and fold-changes of expression in bacterial injection-sample data are shown as purple bars. Bars represent the mean transcript levels ± 95% CI. Means were compared using the unpaired Student’s t-test (* p < 0.05, ** p < 0.01, *** p < 0.001, **** p < 0.0001).

In male bed bugs, the expression of CL-prolixicin1 isoforms in the midgut and RoB varied according to bacteria types that were ingested with blood or injected into the hemocoel. In the midgut, expressions increased ninefold (p < 0.0001) and eightfold (p < 0.0001) after ingestion of *E. coli* and *B. subtilis*, respectively, increased sixfold (p < 0.001) after *E. coli* injection, but did not change after *B. subtilis* injection (Fig. 4a). In the RoB, expression did not change after ingestion of *E. coli*, increased fourfold (p < 0.05) after *B. subtilis* ingestion, and 27-fold (p < 0.0001) and 24-fold (p < 0.0001) after *E. coli* and *B. subtilis* injections, respectively (Fig. 4b).

In the midgut of female bed bugs, the expression of CL-prolixicin 1a &1b increased threefold after ingestion of either Gr– or Gr+ bacteria (p < 0.05 each) and increased 28-fold and 94-fold following injection of Gr– and Gr+ bacteria, respectively (p < 0.0001 each; Fig. 4c). In the RoB of female bed bugs, there was no increase in CL-prolixicin 1a &1b expression following ingestion of blood containing Gr– or Gr+ bacteria, but expression increased 21-fold and 46-fold after injection of Gr– and Gr+ bacteria, respectively (p < 0.0001 each; Fig. 4d).

Gene expression of CL-prolixicin1 in the midgut and RoB was generally higher in female than in male bed bugs, particularly in the RoB in response to all treatments, and in the midgut after *E. coli* and *B. subtilis* injection (Fig. 4e,f). However, the ingestion of *E. coli* and *B. subtilis* caused significantly higher expression of CL-prolixicin1 in the midgut of males than of females (Fig. 4e).

The mode of immune challenge—bacterial ingestion or injection—caused different expression levels of CL-prolixicin1 in male and female bed bugs. In the RoB of males, injection of *E. coli* and *B. subtilis* caused 44.5-fold (p < 0.0001) and 4.6-fold (p > 0.05), respectively, higher expressions of CL-prolixicin1 than did ingestions of *E. coli* or *B. subtilis* (Fig. 4g). In the midgut of males, injections of *E. coli* or *B. subtilis* caused significantly lower expressions of CL-prolixicin1 than did bacterial ingestion (p < 0.0001 each). In the both midgut and RoB of females, injections of *E. coli* or *B. subtilis* caused higher expressions of CL-prolixicin1 than did ingestions of *E. coli* or *B. subtilis* but the effects were statistically significant only for the RoB (*E. coli*: p < 0.001; *B. subtilis*: p < 0.0001; Fig. 4h).

### Distinct gene expression of CL-prolixicin2 in midgut and RoB of male and female bed bugs after bacterial ingestion or injection, and inhibitory effects of CL-prolixicin2 on *T. cruzi* and its motility (Fig. 5)

**Figure 5:**
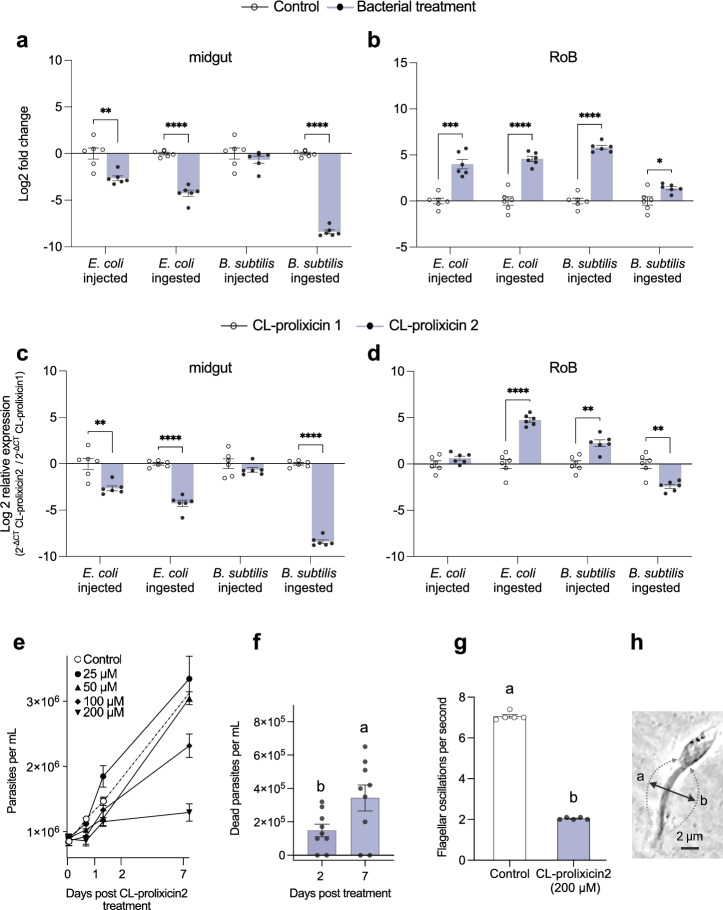
Expression dynamics of CL-prolixicin2 and effects of synthetic CL-prolixicin2 on the parasite *Trypanosoma cruzi*. (a,b) Changes in expression levels of CL-prolixicin2 (LOC122127760) in the midgut and RoB (rest of body containing bodies minus heads and midgut tissues) of male bed bugs 12 h after intrathoracic injection or ingestion of Gram-negative bacteria (*Escherichia coli*) or Gram-positive bacteria (*Bacillus subtilis*). White bars represent data obtained from control bugs that were injected with phosphate buffer saline (PBS), or that ingested sterile blood. The relative expression of CL-prolixicin1a & 1b (LOC106664366) was evaluated using the DDCT method^64,65^. (c,d) Comparison of expression levels of CL-prolixicin1a & 1b (LOC106664366) and CL-prolixicin2 (LOC122127760) in the midgut and RoB of bed bugs. The effect of bacterial ingestion *versus* bacterial injection was compared using the formula 2^−ΔCt^ CL-prolixicin2/2^−ΔCt^ CL-prolixicin1. The ingestion-sample data (white bars) representing the calibrator were arbitrarily set to 1, and fold-changes in CL-prolixicin2 expression in bacterial injection-sample data are shown as purple bars. In all subpanels, bars represent the mean transcript levels ± 95% CI. Means were compared using the unpaired Student’s t-test (* p < 0.05, ** p < 0.01, *** p < 0.001, **** p < 0.0001). (e) Proliferation of *T. cruzi* over the course of 7 days in response to treatment, or not (control), with CL-prolixicin2 at 25–200 µM. (f) Numbers of dead *T. cruzi* on days 2 and 7 as a result of exposure to CL-prolixicin2 at 200 µM; note: there were no dead parasites in the control treatment at days 2 and 7. (g) Flagellar oscillations per second of *T. cruzi* (Y Strain) 7 days after exposure, or not (control), to CL-prolixicin2 at 200 µM. Bars represent the mean transcript levels ± 95% CI. Means were compared using the unpaired Student’s t-test. Different letters above bars in each of subpanels f and g indicate significant differences between samples (p < 0.05). (h) During one complete oscillation, the flagellum moves from “a” to “b” and back to “a”. Per-second oscillations were averaged over 10 s.

In the midgut of male bed bugs, the expression of CL-prolixicin2—unlike the expression of CL-prolixicin1– was significantly down-regulated in almost all treatments (Fig. 5a). However, in the RoB of males, the expression of CL-prolixicin2 increased 26-fold (p < 0.0001) and 2.7-fold (p < 0.05) after *E. coli* and *B. subtilis* ingestion, respectively, and increased 22-fold (p < 0.001) and 60-fold (p < 0.0001) after injection of *E. coli* and *B. subtilis*, respectively (Fig. 5b).

In the midgut of males, the expression of CL-prolixicin1 significantly exceeded that of CL-prolixicin2 in almost all treatments (Fig. 5c). Conversely, in the RoB of males, injection and ingestion of *E. coli,* and injection of *B. subtilis,* increased expression of CL-prolixicin2 1.6-fold, 28-fold (p < 0.0001), and 5.4-fold (p < 0.01) more, respectively, than CL-prolixin1 expression (Fig. 5d). In contrast, ingestion of *B. subtilis* caused a 6.2-fold (p < 0.01) lower expression of CL-prolixicin2 than of CL-prolixicin1 (Fig. 5d).

Synthetic CL-prolixicin2 had a dose-dependent effect on the proliferation, motility, and mortality of *T. cruzi* (Y strain)*.* Over the course of 7 days, synthetic CL-prolixicin2 (200 µM) significantly hindered the proliferation of *T. cruzi* compared to  a control treatment (Fig. 5e). CL-prolixicin2 at 200 µM also caused substantial mortality of *T. cruzi*, with no dead parasites observed in the control or in treatments with < 200-µM CL-prolixicin2 (Fig. 5f). Furthermore, 200 µM of CL-prolixicin2 significantly impaired the motility of *T. cruzi*, with fewer flagellar oscillations per second (recorded at day 7) in treatment than in control parasites (Fig. 5g).

### Activity of synthetic CL-prolxicin2 against bacteria (Table1)

**Table 1:** Minimal inhibitory concentration (MIC) of CL-prolixicin2 against bacterial strains.

	MIC (µM)
Gram-negative bacteria	
* Escherichia coli* Dh5alpha	0.5
* Escherichia coli* KB21	1.0
* Pseudomonas aeruginosa*	> 128
* Serratia marcescens*	16
* Serratia marcescens,* bed bug symbiont	> 128
* Klebsiella pneumoniae*	> 64
Gram-positive bacteria	
* Bacillus subtilis*	32
* Staphylococcus aureus*	> 128
* Staphylococcus sciuri*	> 128
* Staphylococcus epidermidis*	> 128

The minimum inhibitory concentration (MIC) of synthetic CL-prolixicin2 that suppressed the growth of various bacteria is compiled in Table 1. CL-prolixicin2 expressed the strongest inhibition against *E. coli* strains DH5alpha (MIC: 0.5 µM) and KB21 (MIC: 1.0 µM) but there was no inhibitory effect on the Gr– bacteria *Pseudomonas aeruginosa* and *Serratia marcescens* which we isolated from the midgut of bed bugs. Even at a concentration of 128 µM, CL-prolixicin2 had no inhibitory effect on the Gr+ bacteria *Staphylococcus aureus* and *Staphylococcus epidermidis* (Table 1). Interestingly, *Serratia marcescens* which was isolated from bed bugs was more resistant to CL-prolixicin2 (MIC > 128 µM) than other strains of *S. marcescens* tested (MIC: 16 µM).

## Discussion

**Figure 6:**
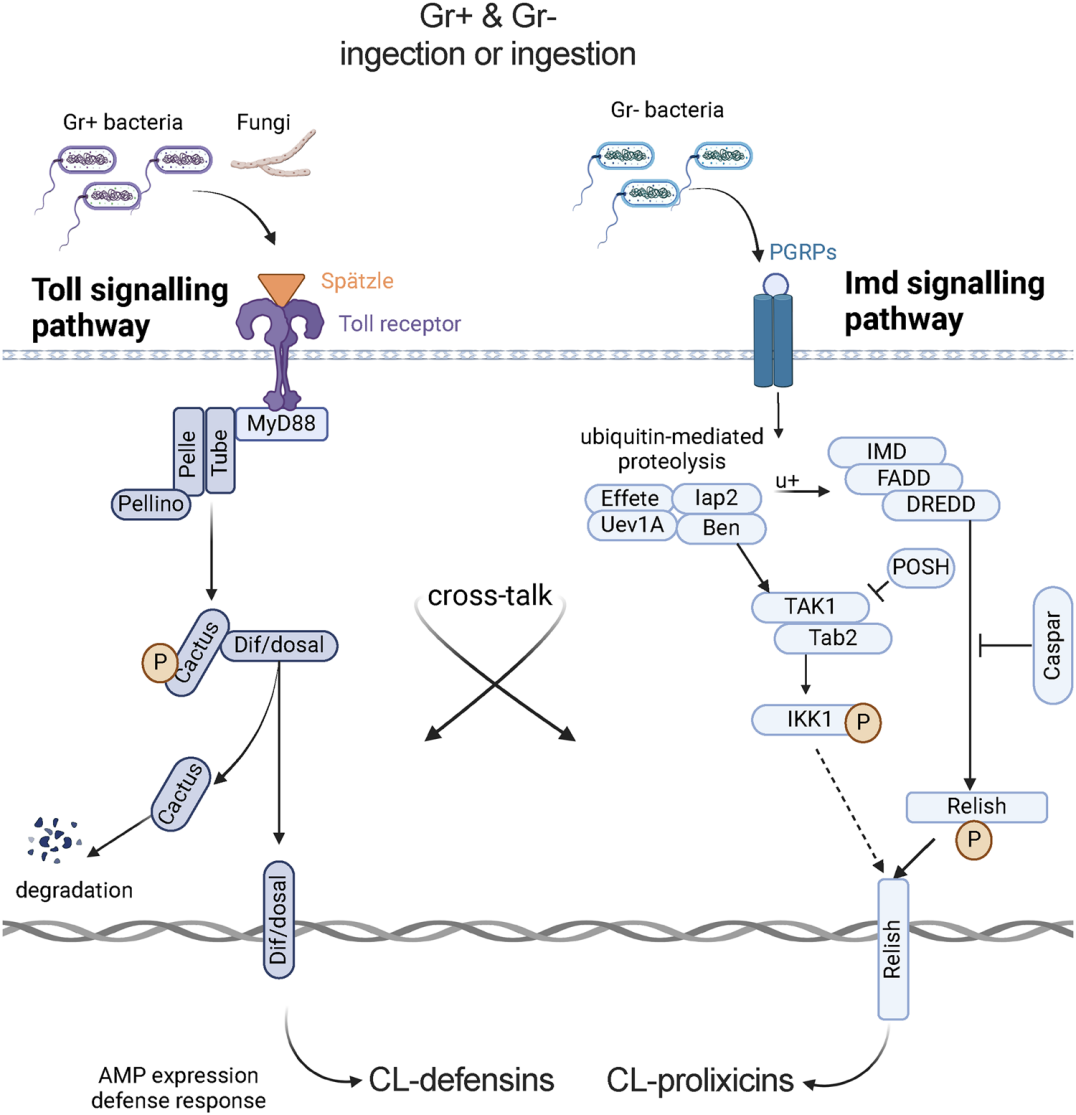
Regulatory cross-talk and functional overlap in Toll and IMD signaling pathways of *Cimex lectularius*. Up-regulation of both CL-defensins and CL-prolixicins in bed bugs in response to the Gram-positive bacterium *Bacillus subtilis* or the Gram-negative bacterium *Escherichia coli* ingestion or injection provides further support for functional cross-talk and blurred functional differentiation between the Toll and the IMD pathways in hemipterans.

We report structural, functional and phylogenetic characteristics of CL-prolixicins and their expression in response to infection with Gr+ and Gr– bacteria. CL-prolixicins show the strongest homology to the prolixicin of *R. prolixus* and have motifs similar to diptericins and attacins. We show up-regulation of CL-prolixicin1 in both the midgut and RoB of bed bugs, and up-regulation of CL-prolixicin2 only in the RoB in response to both Gr+ and Gr– bacteria regardless of the mode of immune challenge. These data suggest that CL-prolixicins may be co-regulated by both IMD and Toll pathways in bed bugs, comparable to the up-regulation of CL-defensins in response to both Gr+ and Gr– bacteria^32^ (Fig. 6). Cross-talk between these pathways has already been demonstrated in other hemipterans^13,41,42^. Intrathoracic injections of Gr+ or Gr– bacteria induced similar up-regulation of the same AMPs^13^, and RNAi of the IMD pathway suppressed the up-regulation of effector molecules in both Toll and IMD pathways^10,13^. Up-regulation of both CL-defensins and CL-prolixicins in bed bugs provides further support for functional cross-talk and blurred functional differentiation between the Toll and the IMD pathways in hemipterans^5,13^. In addition to intracellular cross-talk, Gram-negative-binding proteins may activate the Toll pathway by stimulating the proteolytic cascade that leads to activation of Spaetzle, resulting in the upregulation of AMPs^43–46^.

Homology searches revealed that the mature peptide or active region (not including the signal or pro-peptide) of CL-prolixicins strongly resembles that of diptericins and the prolixicins isolated from *R. prolixus* and other kissing bugs (~ 50% identity, E = 2e−11). In the C-terminus of CL-prolixicins, there was a strong homology to an attacin-C conserved motif (cl04253), strongly suggesting an AMP encoded by this gene. Similar to the prolixicin of *R. prolixus*, CL-prolixicins lack the proline-rich region (P-domain) and the RXRR motif which signals the end of the pro-peptide region. The prolixicin of *R. prolixus* lacks a P-domain and thus was distinguished from the diptericins of *D. melanogaster*^16^. Similarly, CL-prolixicins of bed bugs lack a P-domain and contain fewer prolines than the prolixicin of *R. prolixus*. In silico amino acid sequence analyses revealed that CL-prolixicins are not glycosylated, similar to the prolixicin of *R. prolixus*^16^, but in contrast to glycine-rich glycosylated molecules, such as attacins^16^, CL-prolixicins are not predicted to contain any post-translational modifications. Glycosylation and glycation residues may mediate correct folding and stability of proteins, and glycosylation may stabilize peptides because non-glycosylated proteins degrade quickly^47^. The addition of synthetic glycosylated and non-glycosylated glycine-rich AMPs did not alter the AMP activity of natural peptides^48,49^.

CL-prolixicin1a & 1b have an additional glycine-rich stretch of sequences (E-region) in their C-terminal region (Fig. 1). ‘CAMPR3’ (a database on sequences, structures and signatures of antimicrobial peptides), ‘I4AMP’ (an antimicrobial peptide predictor using physicochemical property-based encoding method and deep learning), and ‘iAMPpred’ (an online prediction server) all predicted antimicrobial activity of the mature protein region of both CL-prolixicins1 and CL-prolixicin2. In contrast, their E-regions were not predicted to have AMP activity. The E-region may have formed due to a duplication event of the G2-domain of CL-prolixicins1a & 1b (Fig. 2). This would be consistent with the observed duplication pattern of attacins^38^. The addition of a P-domain to previous AMPs may have given rise to diptericins in flies, and subsequent gene duplication may have added the second C-terminal glycine-rich motif found in attacins^38^. Close proximity of the bed bugs’ CL-prolixicins on the same chromosome (Fig. 3c) further supports a close functional relationship of these peptides^38^ similar to the close linkage between glycine-rich attacin-A and -B genes observed in *Drosophila*^38^. Diptericins and attacins seem to form a monophyletic group with other AMPs^16,38^.

In bed bugs, CL-prolixicin2 is expressed only in RoB samples, whereas CL-prolixicin1 is expressed in both the midgut and the RoB. In *R. prolixus*, prolixicin is produced in fat body and midgut tissues in response to bacterial infection of the hemolymph or the midgut^16^. The immune competence of the midgut is well characterized in many insects. In *Drosophila* sp.*,* attacins and diptericins are expressed in the digestive tract in response to oral infection with Gr– bacteria^50^. In bed bugs, CL-prolixicin1 is expressed in the midgut and may help eliminate bacteria ingested with blood, analogous to the antibacterial role of the prolixicin in kissing bugs which are close phylogenetic relatives of bed bugs^16^.

Our synthetic CL-prolixicin2 acted more strongly against Gr– bacteria (e.g., *E. coli*) than G+ bacteria. This differential activity aligns with that of other proline- and glycine-rich peptides, such as the prolixicin of *R. prolixus*^16^. Furthermore, CL-prolixicin2 expressed different activity against the *Serratia marcescens* strain isolated from the bed bugs’ midgut than against other bacteria, suggesting that bed bugs may effectively discern between microbes that are potentially harmful to them and those that are obligate symbionts. The 200-µM concentration of CL-prolixicin2 that caused antiparasitic activity against *T. cruzi* is significantly higher than antiparasitic concentrations of potent traditional AMPs such as melittin^51,52^, but it still favorably compares with antiparasitic activity concentrations of other molecules. For instance, the antiparasitic medication benznidazole—which is used against acute infection of humans with *T. cruzi*—expresses activity at about 220 µM. Moreover, MIC values of CL-prolixicin are just one efficacy criterion. CL-prolixicin not only reduced the reproduction and survival of *T. cruzi*, it also severely impaired its motility^51,52^. Antiparasitic activity of various AMPs, such as BatxC, cecropin, crotalicidin, defensin a1138, dermaseptins, and mellitin, against *T. cruzi* has previously been demonstrated under laboratory conditions^16,31, 52–54^. Results obtained in laboratory experiments suggested that *T. cruzi* may survive in the bed bugs’ midgut but is killed if it enters the hemocoel^27,29^. In our study, CL-prolixicn2 was expressed exclusively in the RoB—with no apparent up-regulation in the bed bugs’ midgut—suggesting that CL-prolixicin2 may not affect the development of *T. cruzi* in the midgut of bed bugs, but that it may inhibit *T. cruzi,* if it moves into the hemocoel. The effectiveness of AMPs is contingent upon their concentration and the duration of exposure^16,31, 52–54^. We can calculate the MIC of CL-prolixicin2 in vitro, but we do not yet know the concentration of CL-prolixicin2 normally expressed in the midgut or the hemocoel of bed bugs. Similarly, we do not know if combinations of CL-prolixicin2 and other AMPs might act synergistically to eliminate *T. cruzi,* and if such combinations are effective at concentrations much lower than those of single AMPs. Further investigations are required to understand the implications and effects of CL-prolixicin2 expressed in bed bugs. Moreover, mRNA expression by bed bugs does not assuredly prove the presence of mature peptides which must be demonstrated in future proteomic studies.

We have shown previously that blood-fed female bed bugs have stronger antimicrobial activity than males^33^ and that the mode of immune challenge—bacterial ingestion or injection—affects CL-defensin expressions differently in males and females^32^. Here, we show that bacterial injection causes higher expression levels of CL-prolixicin1 in the midgut and RoB of females than of males, and that females have an overall higher expression of CL-prolixicins than males (Fig. 4). Our data provide support for the hypothesis that the bed bugs’ unusual reproductive strategy of copulatory wounding and traumatic insemination coupled with the risk of microbial infections^34,35, 55^ may cause a more up-regulated immune response in females than in males^34,35, 55^.

The insect immune system comprises constitutively expressed components, and inducible components that are expressed in response to the presence of pathogens. Depending on the immune response that is activated, trade-offs between some aspects of insect immunity and reproductive fitness may occur^56^. In addition, there is a balancing selection for keeping and expressing immune components such as AMPs, and ensuring the well-being of essential bacterial symbionts and microbiota on which many insects rely for survival^57,58^. Bed bugs rely on their *Wolbachia* sp. symbionts to obtain essential nutrients (e.g., B vitamins) not present in vertebrate blood^26,59, 60^. Therefore, there must be mechanisms by which bed bugs eliminate potential pathogens while not reducing essential obligate mutualist symbiont populations to non-effective levels. *Wolbachia sp.* is an intracellular symbiont and as such may be protected from many immune factors circulating in the hemocoel or midgut. Consequently, there emerges a need for the meticulous modulation of the bed bugs' AMPs, ensuring they do not harm the diverse consortium of microbial symbionts. Host–microbe symbioses are predicted to involve adaptive changes in the host immune system to accommodate the microbial partner^61^. The effects of immune responses by bed bugs on their endosymbionts and microbiome, and the fine line between succumbing to bacterial infections, regulating a balanced microbiome, and avoiding elimination of beneficial symbionts have not yet been elucidated.

In conclusion, we have discovered three new glycine-rich antimicrobial peptides in bed bugs which we named CL-prolixicin1a, 1b, and CL-prolixicin2. We analysed their molecular structure and functional characteristics, and studied their phylogenetic relatedness to glycine-rich attacins and diptericins isolated from other insects. We also tested the expression of these CL-prolixicins in midgut and RoB tissues of bed bugs in response to immune challenges. Using comparative transcriptomics and qPCR, we found sex-specific and immune challenge-specific (bacterial ingestion *vs*. bacterial injection) induction of the CL-prolixicins. Synthetic CL-prolixicin2 was not only active against Gr– and G + bacteria but also against *T. cruzi*, the causal agent of Chagas disease. Synthetic CL-prolixicin2 impeded the proliferation of *T. cruzi*, impaired its motility, and caused significant mortality in a dose-dependent manner. These results provide a strong impetus for testing CL-prolixicins for their effects on other parasites and pathogens, including viruses and fungi. With ever-increasing antibiotic drug resistance^31^ and the shortage of new antiparasitic drugs^31^, new AMPs, such as prolixicins in combination with other AMPs, should be evaluated for their potential involvement in therapeutic applications, including the treatment or cure of Chagas disease.

## Methods

### Laboratory rearing of bed bugs

Bed bug colonies were maintained as described previously^62^. Briefly, colonies were kept in the insectary of Simon Fraser University (SFU) at a temperature of ∼24 °C, ambient relative humidity, and a photoperiod of 14 h light to 10 h dark. Groups of 150 bed bugs were maintained in 50-mL glass jars fitted with a square of cardboard (2 cm × 2 cm) at the bottom and a strip of cardboard (2 cm × 4 cm) diagonally across the jar. Bed bugs in separate jars were fed on the forearm of a volunteer (Regine Gries) once every month. For feeding, jars were covered with fine mesh, inverted, and pressed against the forearm so that the bed bugs could feed through the mesh.

### Growth and preparation of bacteria for immune challenges of bed bugs

*Escherichia coli* (K12/D31) and *Bacillus subtilis* (ATCC 6633**)** were grown in separate Lysogeny Broth (LB)^63^ for 17 h at 30 °C in a shaking incubator (220 revolutions per minute). Subsequently, the bacteria were reinoculated into fresh broth and incubated 4 h under the same conditions to reach the log phase of growth. The bacteria were then washed three times in sterile phosphate-buffered saline (PBS; 0.01 M phosphate buffer, 2.7 mM potassium chloride, 0.137 M sodium chloride, pH 7.4), and were diluted either in sterile defibrinated rabbit blood or in PBS to a final concentration of ~ 1 × 10^6^ cells/mL.

Bed bugs either ingested microbe-laced blood or received an intrathoracic (hemocoelic) bacterial injection. For ingestion of microbes, we allowed male and female bed bugs (food-deprived > 20 days) to feed 1 h on defibrinated, microbe-laced (*E. coli* or *B. subtilis*) rabbit blood in a water-jacketed membrane feeder (Thermo Fisher Scientific Isotemp 2150 B14, USA) set to 37 °C, with stretched out parafilm as the membrane. Control bed bugs ingested sterile blood. Fully engorged bed bugs were separated and housed in glass jars until analysis. For intrathoracic injections, 0.5 μL of a bacterial broth (*E. coli*, *B. subtilis*, or both at a final concentration of approximately 1 × 10^6^ cells/mL), or a PBS control, were injected into the hemocoel of > 20-day food-deprived male or female bed bugs. Each experiment had 5–10 replicates, with five insects each in treatment and control samples.

### Tissue isolation and RNA extraction

Midgut and RoB tissues of treatment and control bed bugs were dissected 12 h after the immune challenge, and total RNA was extracted using TRizol reagent (Invitrogen) following the manufacturer’s recommendations. The samples were quantified on a Nanodrop 1000 spectrophotometer v. 3.7 (Thermo Fisher Scientific, USA). RNA samples were used to generate cDNAs for analysis using qPCR and transcriptome assembly. The methodologies employed for cDNA Synthesis and Quantitative Real-Time PCR (qPCR), as well as Transcriptome Assembly, including library preparation with polyA selection, HiSeq sequencing, and RNA-Seq data analysis in this manuscript, are consistent with the protocols established in our preceding study with minor modifications, reinforcing the reliability and reproducibility of our results^32^.

### cDNA Synthesis and Quantitative Real-Time PCR (qPCR)

For this section, we adhere to our previous study, with slight modifications^32^. First strand cDNA synthesis was performed in 20-μL reaction mixtures containing 2.0 μg total RNA using a modified oligo dT primer with the OneScript cDNA Synthesis Kit (ABM, CA) and an extension time of 50 min. The subsequent cDNA was diluted (1:10) with sterile molecular grade RNase-free water. The expression of transcripts of CL-prolixicins in the different samples was assessed using qPCR. All qPCR reaction mixtures contained 2 μL of cDNA, 300 μM of each primer, and 5 μL of PerfeCTa SYBR Green Super Mix (Quanta Biosciences, USA) in a final volume of 10 μL. qPCR was performed on a LightCycler96 thermal cycler (Roche Diagnostics, DE), with the following conditions: 95 °C (5 min), 40 cycles each at 95 °C (15 s) and 60 °C (40 s), followed by a melt curve analysis to confirm the specificity of reactions. No-template controls were included with each primer set to verify the absence of exogenous DNA and primer-dimers. Each primer pair had 99% efficiency as determined using the slope of a linear regression model (SM, unpubl. data). The primer sequences are reported in Supplemental Table S1. Primers were designed or retrieved from the literature for ribosomal protein (RPL18) as the internal control gene, which provides the most stable gene expression across all tissues and developmental stages of bed bugs, and CL-prolixicins. The amplicons were sequenced to confirm they had amplified the correct sequence.

Relative differences in transcript levels were calculated using the DELTA (Δ) threshold cycle (CT) method 2^–ΔΔCT^^64,65^. We normalized expression levels using an internal control gene (RPL18) and to generate ΔCT values. In the 2^−ΔΔCT^ method, we used control samples (bed bugs injected with a PBS control or fed sterile blood) as the second calibrator to measure fold changes in expression levels of CL-prolixicin1a & b mRNA, and CL-prolixicin2 mRNA (Figs. 4, 5). We compared bed bug sex-dependent gene expression of CL-prolixicin1a & b mRNA, using the formula 2^−ΔCT^ female/2^−ΔCt^ male (Fig. 4e,f), and we compared the effects of intrathoracic bacterial injection and oral bacterial ingestion, using the formula 2^−ΔCt^ injected/2^−ΔCt^ ingested (Fig. 4g,h). The results are presented as means and standard errors of at least three independently generated cDNAs assayed at least twice, with each sample run in three technical replicates. All data sets were tested for normality using the Shapiro–Wilk normality test, and were compared using the unpaired Student’s *t*-test or Mann–Whitney U test, when appropriate. Prism version 9.4.1 software (GraphPad Software, San Diego, CA, USA) was used for statistical analyses and 2D graphing. The statistical significance level was 0.05. Relative transcript levels are expressed as means with whiskers representing ± SEM (* *p* < 0.05, ** *p* < 0.01, *** *p* < 0.001, **** *p* < 0.0001).

### Transcriptome assembly: library preparation with polyA selection and HiSeq sequencing and RNA-Seq data analysis

This section updates our previous study, with minor alterations^32^. We created a de novo transcriptome assembly from RNA extracted from midgut and RoB tissues of male bed bugs 12 h after ingesting blood containing *E. coli* or *B. subtilis*. RNA purification, first and second strand synthesis, adaptor ligation, quantification, validation, and Illumina sequencing were all done at GENEWIZ LLC. (South Plainfield, NJ, USA).

The RNA samples were sequenced using a paired-end (PE) configuration consisting of two 150 base pair (bp) strands. HiSeq Control Software (HCS) was used for image analysis and base calling. Raw sequence data (.bcl files) generated from Illumina HiSeq were converted into fastq files and demultiplexed with bcl2fastq 2.17 software (Illumina Inc, San Diego, CA, USA). For index sequence identification, one mismatch was allowed. After examining the raw data quality, adapter sequences and nucleotides of poor quality were removed from sequence reads using Trimmomatic v.0.36. The trimmed reads were mapped to the reference genome available on ENSEMBL using STAR aligner v.2.5.2b which utilizes a splice aligner to detect splice junctions and incorporates them to help align the entire read sequences to generate Binary Alignment Map (BAM) files. In the Subread package version 1.5.2, we used the ‘Counts’ feature to calculate the unique gene count. Only reads that fell within exons were counted.

We used the gene hit counts table for downstream differential expression analysis, using DESeq2^66^ to compare gene expression between groups of samples. We used the Wald’s test to generate *p*-values and Log2 fold-changes. We considered genes to be differentially expressed when adjusted *p*-values were < 0.05 and absolute log2 fold-changes were > 1. The data for prolixicin transcripts were mined to evaluate their expression level, and are reported as transcripts per million (TPM).

### Prolixicin characterization

The full-length peptide sequences of bed bug prolixicins were retrieved from https://www.ncbi.nlm.nih.gov/. All prolixicin amino acid sequences were submitted to the SignalP6.0 server (https://services.healthtech.dtu.dk/services/SignalP-6.0/) to predict signal-peptides. Pro-peptides were detected by the ProP 1.0 Server (http://www.cbs.dtu.dk/services/ProP/). The net charge of the mature region of prolixicins at pH 7 and their molecular weights were predicted at PROTEIN CALCULATOR v3.4 server (http://protcalc.sourceforge.net/). The potential antimicrobial properties of the sequences were predicted in the Collection of the Anti-Microbial Peptides (CAMP_R3_) server (http://www.camp.bicnirrh.res.in/prediction.php), in ‘I4AMP’ (an antimicrobial peptide predictor using physicochemical property-based encoding method and deep learning), and in ‘iAMPpred’ (an online prediction server) (Supplemental Table S2). Sequence similarity searches for obtained sequences were run with BLAST, using the database of NCBI (http://blast.ncbi.nlm.nih.gov/Blast.cgi). Tertiary structures of mature or active prolixicin peptides (Fig. 3a,b) were predicted by iTASSER (https://zhanggroup.org/I-TASSER/) and AlphaFold2, and tertiary structure models were visualized using UCSF Chimera^67^. In silico analyses to identify potential sites for post-translational modifications, including glycosylation and phosphorylation, were performed using tools from the Center for Biological Sequence Analysis (http://www.cbs.dtu.dk/services/).

### Phylogenetic analysis

Multiple complete sequences, or conserved domain sequences, were aligned with MUSCLE aligner (https://www.ebi.ac.uk/Tools/msa/muscle/) using Jalview^68^. Alignments were used to build phylogenetic trees using iqtree-2.0-rc2 by selecting the best-fit substitution models PMB + F + G4 (Fig. 2). Maximum likelihood analyses were done using IQ-TREE v. 2.0^69^. Best-fit models for each alignment were selected based on Bayesian information testing, including 10,000 replicates of Ultrafast bootstrap (UFBoot) to provide support for tree branches. In our study, both BLOSUM62 and PMB models generated very similar results with good agreement. The tree presented here was prepared by FigTree software v. 1.4.4 (http://tree.bio.ed.ac.uk/software/figtree). For phylogenetic tree construction, different domains (N-terminal domain, G1- and G2-domains) of prolixicins from bed bugs, *R. prolixus*, and other insects were utilized. *Lasioderma serricorne* N-domain was used as an outgroup for the phylogenetic tree construction to provide a point of reference for rooting the tree and determining the evolutionary relationships among the studied prolixicin domains.

### Synthesis of CL-prolixicin2

The CL-prolixicin2 peptide tested in our study was custom-synthesized by Biomatik Corp. (Cambridge, ON N3H 4R7, CA). Analysis of the synthetic peptide by coupled high-performance liquid chromatography—mass spectrometry (HPLC–MS) revealed a peptide purity of > 95% (Biomatik).

### Antimicrobial activity of CL-prolixicin2

Minimal inhibitory concentrations (MICs) of synthetic CL-prolixicin2 were determined using Mueller Hinton (MH) broth microdilutions in 96-well microtiter plates in accordance with guidelines of the Clinical and Laboratory Standards Institute (CLSI), and the standard two-fold broth microdilution method^70^, with minor modifications. Briefly, bacteria were grown overnight in MH broth for 17 h at 30 °C in a shaking incubator (220 revolutions per minute). Subsequently, the bacteria were reinoculated into fresh broth and incubated 4 h under the same conditions to reach the log phase of growth and then diluted to give a final inoculum size of 10^5^ CFU/mL. Peptides were serially diluted with MH broth to concentrations of 0.0078 µM to 128 µM (2-times serial dilution) in 96-well microtiter plates (Nunc Multiwell Plates; Thermo Fischer Scientific, CA) at 10 μL/well. Bacterial inoculum was added to each well (90 μL) and incubated 20 h at 30 °C. MICs were identified as the lowest concentration of CL-prolixin2 that inhibited bacterial growth as determined by measuring the optical density at 600 nm, using a microplate reader (Shimadzu UV-2550 spectrophotometer; Sognsveien 70 A, 0855 Oslo, NO). The following bacteria were tested: Gr–: *Escherichia coli* Dh5alpha, *Escherichia coli* BL21, *Pseudomonas aeruginosa*, *Serratia marcescens*, *Serratia marcescens* isolated from the anterior midgut of bed bugs, and *Klebsiella pneumonia*; Gr+: *Bacillus subtilis*, *Staphylococcus aureus*, *Staphylococcus sciuri*, and *Staphylococcus epidermidis*. The selection of these strains was based on their clinical and environmental relevance, as well as their divergent susceptibility to antimicrobial agents, thereby providing a comprehensive assessment of the antimicrobial activity expressed by synthetic CL-prolixicin2. The positive control consisted of a bacterial culture (*E. coli* BL21) and of ampicillin (antibiotic in penicillin family) across a similar concentration range, and the negative control was MH broth alone. Each experimental condition was run in triplicate across three biological replicates to ensure the reliability and reproducibility of the results.

### Effect of CL-prolixicin2 on the viability of *T. cruzi* and on its flagellar movements

*Trypanosoma cruzi* strain Y (ATCC Chagas 50832™; obtained from Cedarlane, Burlington, ON, CA), previously isolated from a Chagas disease patient in Brazil, was cultured in LIT (liver infusion tryptose) medium at 25 °C. Parasites in the mid-logarithmic growth phase were adjusted to a density of 1 × 10^6^ parasites/mL for experimentation.

Synthetic CL-prolixicin2 was serially diluted (twofold dilution) to concentrations of 25 µM to 200 µM in 96-well microtiter plates (Nunc; Thermo Fischer Scientific) at 20 μL/well. Aliquots (180 μL) of the parasite culture (1 × 10^6^ parasites/mL) were added to each well and incubated at 25 °C. At days 1, 2 and 7 post incubation, live or dead flagellated parasites were counted in a Neubauer chamber, using an optic microscope (Carl Zeiss Inc., White Plains, NY, USA). Following treatment, the viability of *T. cruzi* was determined by counting live flagellated trypomastigotes and epimastigotes (developmental stages of *T. cruzi*) using a hemocytometer. Parasites were classed as ‘alive’ based on their motility. Live parasites were counted in nine samples treated with CL-prolixicin2 and in nine control samples. Results are reported as the number of live or dead parasites per mL of parasite culture.

The effect of CL-prolixicin2 (200 µM) on the motility of live flagellated trypomastigotes and epimastigotes was assessed seven days post incubation. Parasites were observed under a Zeiss microscope with phase contrast at 400× magnification, and their flagellar oscillations were recorded in slow motion using an AxioCam MRm camera connected to AxioVision software (Version 4.8.2, Carl Zeiss Inc.). Per-second oscillations (back-and-forth movements) of the flagellum (Fig. 5h) were counted and averaged over 10 s. Data were collected from 10 randomly selected parasites in each of the five replicates.

Data were analyzed by a t-test or one-way ANOVA with Tukey's multiple comparison test when appropriate to identify significant differences between control groups and CL-prolixicin2-treated groups of parasites. Prism version 9.4.1 software (GraphPad Software, San Diego, CA, USA) was used for statistical analyses and 2D graphing. The statistical significance level was 0.05.

### Supplementary Information

Supplementary Table S1.

Supplementary Table S2.

## Data Availability

The datasets generated and/or analysed during the current study are available in the NCBI Gene Expression Omnibus (GEO) repository under the accession number GSE256026.
